# New functions of DDR1 collagen receptor in tumor dormancy, immune exclusion and therapeutic resistance

**DOI:** 10.3389/fonc.2022.956926

**Published:** 2022-07-22

**Authors:** Audrey Sirvent, Kevin Espie, Evangelia Papadopoulou, Dana Naim, Serge Roche

**Affiliations:** Centre de Recherche en Biologie cellulaire de Montpellier (CRBM), Centre National de la Recherche Scientifique (CNRS), Univ. Montpellier, Montpellier, France

**Keywords:** Keywords: collagen, tumor microenvironment, receptor, tyrosine kinase, immune evasion, metastasis, tumor dormancy, therapeutic resistance

## Abstract

The tumor microenvironment facilitates cancer progression and therapeutic resistance. Tumor collagens and their architecture play an essential role in this process. However, little is known about the mechanisms by which tumor cells sense and respond to this extracellular matrix environment. Recently, the Discoidin Domain Receptor 1 (DDR1), a collagen receptor and tyrosine kinase has emerged as an important player in this malignant process, although the underlying signaling mechanisms remain unclear. Here, we review new DDR1 functions in tumor dormancy following dissemination, immune exclusion and therapeutic resistance induced by stromal collagens deposition. We also discuss the signaling mechanisms behind these tumor activities and the therapeutic strategies aiming at targeting these collagens-dependent tumor responses.

## Tumor collagens

Extracellular matrix (ECM) components from the tumor microenvironment (TME) play an important role in epithelial tumor development (1, 2). Specifically, aberrant deposition of collagens, the most abundant components of tumor ECM, defines a bad prognosis marker in several cancer patients and contributes to their tumor progression (3). TME collagens are secreted by cancer-associated fibroblasts (CAF), tumor-associated macrophages (TAM) and tumor cells themselves, and are deposited within or around the tumor and at the metastatic site. They are modified by components secreted by tumor and stromal cells in the TME that modulate their organization, resulting in wavy or fibrotic and aligned fibers. For instance, TME acidification and secretion of metalloproteases, collagenases or lysine oxidases induce cross-linkage and stabilization of insoluble collagen deposited in tumor tissues (4, 5). Other post-translational modifications may participate in collagens’ organization locally, as reported for Peptidyl Arginine Deaminase activity that promotes dense collagen fibers at the metastatic niche (6). Besides the nature of TME collagens, these architectures define important prognostic markers of the disease. Consistent with this notion, deposition of a dense and reticulated collagen induces tumor stiffness, resulting in enhanced tumor growth, reduced immune infiltration, and promote metastatic colonization (4, 5).

## The collagen receptor DDR1

The mechanism by which tumor cells respond to the heterogeneous collagen-enriched microenvironment is not clearly established. Collagens bind to 6 receptor types (i.e Integrins, DDRs, Lairs, GPVI receptors, and OSCAR) (7). While integrins may play a role in this process (8), several of these tumor responses also implicate DDR1 (9, 10). DDR1 belongs to the DDRs family of the receptor tyrosine kinase (RTK), which includes DDR1 and DDR2 (11, 12). DDRs exhibit a similar modular structure to RTKs (*i.e.* extracellular domain with ligand binding, short transmembrane domain, and large cytoplasmic tail containing a kinase domain); however, unlike other RTKs,DDRs bind to the ECM collagens, and theirr kinase activation kinetic is slow but sustained over time (11, 12). Although the underlying mechanism is not fully understood, DDR1 regulation involves its aggregation into large clusters where collagen induces the lateral association of receptor dimers (*i.e*. receptor clustering) and phosphorylation between dimers (13–15). DDR1 is activated by most collagen types, including I and IV, which are abundant in the basement membrane (11, 12). DDR1 is preferentially expressed in epithelial tissues and acts as a cellular sensor of the collagen-enriched microenvironment by modulating growth and adhesive receptors signaling (12). In physiological conditions, *DDR1* deficient mice revealed an important function of DDR1 in mammary gland development as well as pancreatic and kidney tissue homeostasis (16–21). Whether DDR1 displays similar function in other epithelial tissues is unknown. DDR1 is upregulated in many epithelial cancers and plays important roles during tumor development. Positive and negative functions were reported depending on the cancer type, the tumor stage and the nature/organization of collagens matrix (11, 12, 22–24). DDR1 has been involved in tumor growth, dissemination and metastasis development, implicating an interplay between DDR1 signaling and collagen remodeling, and these functions have been extensively discussed in previous reviews (11, 12, 22–24). Here we discuss recent findings uncovering additional DDR1 functions in tumor dormancy following dissemination, immune exclusion and therapeutic resistance governed by specific TME collagen-enriched matrices.

## DDR1 in cancer dormancy and metastatic reactivation

Metastatic development is an inefficient multistep process characterized by local tumor cell invasion of the stroma surrounding the tumor, followed by dissemination in the blood or lymphatic circulation and colonization of distant tissues (25, 26). While metastatic development is initiated by disseminated tumor cells (DTCs) with cancer stem cell properties, this whole process is supported by the aberrant tumor cell communication with their microenvironment (i.e. the front of the primary tumor, the stroma, the blood vessels and the metastatic niche of the colonized tissue) (25, 26). In this context, aberrant collagen deposition plays an essential role throughout this malignant process and DDR1 contributes to tumor cell responses to these collagen-enriched microenvironments. For instance, DDR1 promotes local invasion of epithelial cancer cells into collagen fibers deposited at the front of the tumor, both in a single or collective mode (27, 28). DDR1 is also essential for homing and colonization of epithelial cancer in the lungs and the bones (10, 29). Similarly, DDR1 upregulation enables liver metastatis of colon cancer cells (30). This metastatic function may be activated by the collagens deposited at the metastatic site from CAFs, TAMs, DTCs and cancer exosomes secereted by the primary tumor (23).

**Figure 1:**
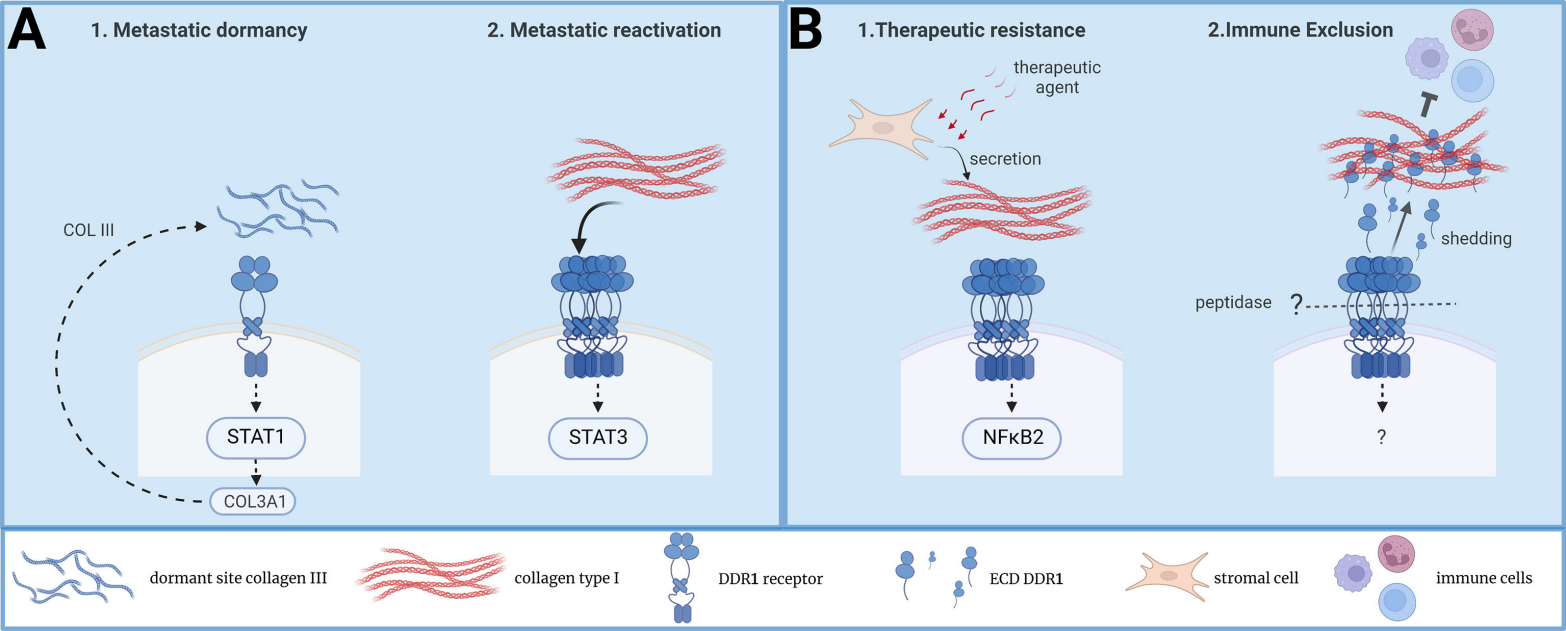
New functions of DDR1 collagen receptor in tumor dormancy, immune exclusion and therapeutic resistance. **(A)** A model on DDR1 signaling during DTC dormancy and metastatic reactivation induced by TME collagens**. (B)** A model on DDR1 signaling during therapeutic resistance and immune exclusion induced TME collagens.

Recently, Di Martino uncovered an additional important function of DDR1 in this metastatic process, through the maintenance of DTCs in a quiescence state (31) (**Figure 1A**). It is known that tumor cell dissemination can occur both at early stages of tumorigenesis (i.e. when the primary tumor is not macrosopically detectable) and during cancer progression (26). These DTCs become non-proliferative (i.e. dormant state) at the metastatic site for long periods of time and are reactivated by specific signals to induce metastatic development (26). This process is at the origin of the clinical manifestation where patients develop metastasis years after the removal of the primary tumor by surgery and chemotherapy. The authors assessed the contribution of local ECM components in the induction of this cellular dormant state by using well-established dormant and proliferative models of human head and neck squamous cacrinoma cells and murine mammary tumor cell-lines in mouse xenograft assays. By comparing collagens’ architecture and composition in both situations, they found a deposition of wavy collagens (i.e. with low degree of linear orientation) enriched in collagen III surrounding dormant tumor cells and showed that this ECM impacts their non-proliferative state. In sharp contrat, proliferative DTCs were surrounded by an extracellular matrix enriched in ECM glycoproteins with aligned collagen fibers. Interestingly, collagen III was provided by landed DTCs, suggesting a self-protective non-proliferative program. Next, the authors demonstrated that DDR1 mediates this collagen III-dependent dormant state. Interestingly, DDR1 dormant signaling was dependent upon its binding to collagen III but independent of its kinase activity. Importantly, they identified a feedforward loop where DDR1 induces *COLA31* expression by a Stat1-dependent mechanism. From this, they propose that *COL3A1* expression remodels the ECM in a wavy collagen architecture to maintain the cells into a dormant state (**Figure 1A**). In agreement with this idea, DDR1 downregulation reactivated DTCs proliferation. Of note, the authors observed that DDR1 was also essential for survival of proliferative DTCs, consistent with a DDR1 function also during metastatic reactivation (10). In this context, DDR1 survival signaling was kinase-dependent and involved Stat3 signaling. Collectively these results support a model where DDR1 regulates both cell dormancy and metastatic reactivation, depending on the architecture of the ECM deposited at the metastatic niche (collagen III-enriched wavy ECM versus collagen I-enriched linear ECM) (**Figure 1A**).

## DDR1 in tumor immune exclusion

Immune evasion is an important feature of aggressive tumors and the ECM deposited at the TME contributes to this pathogenic effect (5). While a dense ECM network surrounding the tumor may prevent immune infiltration (5), the underlying mechanism is largely unclear. Recently, Sun et al. reported a central role of DDR1 in this malignant process (32) (**Figure 1B**). By using murine models of mammary tumor cells, they could show that DDR1 ablation prevented tumor development in immuno-competent, but not in immune-deficient mice, thus uncovering a new DDR1 function in tumor immune evasion. This effect was attributed to an immune exclusion effect rather than an inhibition of infiltrated cytotoxic lymphocytes. Importantly, the authors reported an inverse correlation between DDR1 tumor expression and CD8+ T cells intra-tumoral levels in a cohort of breast cancer patients, highlighting the clinical relevance of their findings. While transcriptomic analysis were not very insightful, functional rescue assays uncovered an unsuspected mechanism behind this new DDR1 tumor function. It is known that DDR1 can be subjected to membrane shedding leading to the release of the whole extracellular domain (ECD-DDR1) (12). Surprisingly, expression of ECD-DDR1 alone was sufficient to promote substantial tumor immune exclusion. Mechanistically, this response was associated with a higher order of dense collagen fibers deposited at the ECM, suggestive of ECD-DDR1 function in ECM remodeling. This hypothesis was next validated by showing that ECD-DDR1, in its multimeric form, efficiently remodels collagen fibers and reduces T cells motility in culture. *In vivo*, injection of purified ECD-DDR1 reduced tumor immune infiltration in tumor xenografts. Finally, they could validate DDR1 as a therapeutic target for tumor immunotherapy by generating a blocking anti-DDR1 antibody. This antibody promoted immune infiltration and reduced tumor development *in vivo*. Unlike tumor xenograft animals, *DDR1* knock-out in genetically modified MMTV-PyMT mice promotes spontaneous mammary tumor development, consistent with a DDR1 negative function during tumor initiation (33). Still, their DDR1 antibody gave a similar anti-tumor effect, suggesting that DDR1 may have a tumor-promoting function at later stages of tumor development. This novel study further confirms previous results showing a function for DDR1 in tumor evasion and resistance to anti-Programmed cell Death protein 1 (PD1) immunotherapy (34). However, it uncovers an additional mechanism by which DDR1 contributes to immune evasion, i.e. the remodeling of TME collagen-enriched matrix unfavorable of immune infiltration. Whether this mechanism is conserved in other epithelial cancers is however unknown. Overall, this study further highlights the important role of DDR1 in tumor immune evasion (**Figure 1B**).

## DDR1 in induced therapeutic tumor resistance

Tumor cells have a remarkable capacity to adapt to different environments and to cellular stresses (e.g. oxidative, oncogenic, and therapeutic) (2). This cellular plasticity is at the origin of the clinical manifestation of cancer patients that become resistant to therapy, resulting in treatment failure. While tumor adaptation can be induced by therapeutic resistant mutations in tumor cells, it can be induced by genetic-independent mechanisms, which involve the TME (3). In this context, Berestjuk et al. reported a central role of DDR1 and DDR2 in this adaptation process (35) (**Figure 1B**). By addressing the influence of TME ECM in the induced resistance of melanoma cells to anti-BRAFV600E therapy, they could show that the ECM produced by CAFs confers a drug-protective action to melanoma cells in culture. This drug tolerance effect was attributed to the aligned and dense collagen I fibers. The authors noticed a high expression of DDR1 and DDR2 in melanoma that was associated with a bad prognosis. Additionally, they associated DDR1/2 expression with melanoma progression and with the invasive and therapy-resistant phenotype. From this, they hypothesized that DDRs would mediate this drug resistance response. Consistently, interaction of melanoma cells with CAFs’ ECMs induces activation and linear clustering of DDR1/2. Functionally, inhibition of DDR1/2 expression or activity overcome ECM-mediated melanoma cell resistance to the targeted therapy *in vitro*. *In vivo*, mice treatment with imatinib, a tyrosine kinase inhibitor (TKi) known to target DDR1/2 activity (36, 37), improved anti-BRAFV600E drug efficacy and delayed tumor relapse. Interestingly, this anti-tumor effect was associated with a diminution of drug-induced collagen remodeling, suggestive of a feedforward loop between DDR1/2 and collagens. However, additional imatinib drug targets (e.g. stromal PDGFR and DDR2) may contribute to the normalization of collagen deposition. Finally, while MAPK signaling was not modified by this resistance mechanism, proteomic analysis pointed to a pro-survival NIK/IKKα/NF-κB2 pathway downstream this DDR1/2 drug resistance effect (**Figure 1B**). Interestingly, this study suggest that DDR1 and DDR2 can signal in a concerted fashion. While the underlying mechanism is unclear, it may likely involve receptor hetero-dimerization. This new DDR1 activity was corroborated in KRAS-mutant lung cancer upon treatment with platinum (38). In this study, DDR1 was found upregulated during chemotherapy and its expression levels correlated with poor response to chemotherapy in lung cancer patients. Importantly, pharmacological DDR1 inhibition gave a synergistic effect with chemotherapy in a number of lung cancer experimental models tested. Whether this DDR1 function was induced by collagen remodeling during therapeutic treatment was not investigated. However, the authors discussed a tetraspanin TM4SF1-dependent mechanism of DDR1 upregulation, previously reported in lung cancer metastasis (39). In summary, these new studies confirm an important function of DDR1 in tumor adaptation to therapy, which implicates TME ECM remodeling.

## Discussion and future direction

These recent findings bring new insights into the complex signaling mechanisms behind DDR1 tumor activities. Both kinase-dependent and independent DDR1 tumor signaling were reported, although their underlying mechanisms remain unclear (12, 23). Interestingly the work of Di Martino suggests that the nature of collagen-enriched ECM (wavy versus dense aligned collagen) would discriminate between DDR1 kinase-independent dormant versus kinase-dependent survival signaling. Specifically, aligned collagen fibers would promote high DDR1 clustering, which is necessary for DDR1 kinase activation (13). However, it is likely that additional factors (ECM, cytokine, receptors) may be involved in this process. Consistent with this idea, in resistant melanoma cells, DDR2 may contribute to DDR1 clustering, enabling kinase-dependent signaling. Similarly, TM4SF1 may act as a co-receptor for DDR1 function during metastatic reactivation (10). While TM4SF1 also induces DDR1 clustering, receptor phospho-signaling was relayed by cytoplasmic protein kinases, such as PKCalpha/JAK2 in metastatic breast cancer (10) and PKCtheta/SYK in metastatic pancreatic cancer (40). These new findings suggest that the combination of collagen-induced DDR1 clustering and their association with specific co-receptors may influence DDR1 phospho-signaling. Sun et al. provided an additional kinase-independent DDR1 signaling mechanism in tumor immune exclusion (32). This activity is mediated by receptor shedding, previously identified as a negative mechanism of DDR1 signaling (12). Importantly, this study brings the first pro-tumoral function of this known molecular process. However, this attractive model raises several important mechanistic questions. For instance, it is unclear whether the endogenous level of ECD-DDR1 produced from the tumor is consistent with its remodeling effect observed in overexpressing system. It is likely that additional factors may contribute to this response. Similarly, additional DDR1 signaling may contribute to this tumor effect and the activity of a shedding-resistant DDR1 allele would be informative in this regard. While ADAM10 and MMPs (41, 42) were involved in DDR1 shedding, the nature of proteases triggering this new DDR1 activity was not addressed, while of potential therapeutic interest. Finally, this model does not address how receptor shedding would cope with its cytoplasmic signaling capacity to mediate collagen DDR1 pro-tumoral activities. For instance, a role of DDR1 was reported in mechanical remodelling of collagen I by tumor cells, which depends on DDR1 clustering and association with myosin (14, 43). It is thus likely that this signaling mechanism also participates in this pathological effect. In this scenario, the shredded DDR1-ECD would be left on collagens after cells reorganize it and would contribute to the DDR1 tumor function reported by Sun et al. (32). Clearly, further investigation is needed in the future to decipher these complex DDR1 tumor signaling mechanisms.

Finally, these recent reports may have important therapeutic consequences on the design of DDR1-based cancer therapies. First of all, they confirm the interest of therapeutically targeting DDR1 to reduce tumor immune evasion, metastatic development and thepareutic resistance in several cancer types. Second, these observations may help in optimising a strategy to chemically inhibit these diverse DDR1 pathogenic activities. For instance, a tumor with dense and aligned collagen fibers could be predictive of DDR1-kinase dependent signaling that could be targeted by TKi. While specific DDR1 kinase inhibitors did not reach the clinic yet, several clinically available TKi (e.g. imatinib, nilotinib, dasatinib) were shown to effectively inhibit DDR1/2 (22) and could be used for this purpose. However, this strategy may not be effective on kinase-independent DDR1 tumor functions. A complementary approach would involve an anti-DDR1 immunotherapy, as demonstrated in experimental settings addressing tumor immune evasion (32, 34). While this approach would target both kinase-dependent and independent DDR1 functions, several adverse effects could be predicted, such as metastatic reactivation of dormant DTCs. Besides, not all tumor-types may be dependent upon TME collagens to mediate immune evasion. For instance, in pancreatic cancer, TME collagen depletion from CAFs led to an increased tumor immune supression consistent with anti-tumor function of TME collagens (44). Whether DDR1 has a role in this anti-tumor effect is currently unknown. Clearly, further investigation is needed to clarify the role of TME collagens and its receptor DDR1 in cancer agressiveness.

In conclusion, these new studies add additional important DDR1 functions in cancer development and confirm this receptor as an attractive target in oncology. Mechanistic insight into these new functions may help to develop effective DDR1-based therapy in order to improve anti-tumor effects of the currently used therapy. Finally, additional exciting new findings on DDR1 tumor function may be expected in the future, such as tumor cell metabolism, another hallmark of cancer (1, 2) influenced by TME collagens (45).

## Author Contributions

SR drafted the first version of the manuscript and KE and EP the figure. All the authors have critically reviewed and approved the manuscript.

## Funding

This work was supported by the Association pour la Recherche contre le Cancer ARC, Montpellier SIRIC Grant «INCa-DGOS-Inserm 6045», CNRS, and the University of Montpellier. SR is an INSERM investigator.

## Conflict of Interest

The authors declare that the research was conducted in the absence of any commercial or financial relationships that could be construed as a potential conflict of interest.

## Publisher’s Note

All claims expressed in this article are solely those of the authors and do not necessarily represent those of their affiliated organizations, or those of the publisher, the editors and the reviewers. Any product that may be evaluated in this article, or claim that may be made by its manufacturer, is not guaranteed or endorsed by the publisher.
